# Unexpected genetic differentiation between recently recolonized populations of a long-lived and highly vagile marine mammal

**DOI:** 10.1002/ece3.732

**Published:** 2013-09-08

**Authors:** Carolina A Bonin, Michael E Goebel, Jaume Forcada, Ronald S Burton, Joseph I Hoffman

**Affiliations:** 1 Center for Marine Biodiversity and Conservation, Scripps Institution of Oceanography, University of California San Diego9500 Gilman Dr., La Jolla, California, 92093-0208; 2 Antarctic Ecosystem Research Division, Southwest Fisheries Science Center, National Marine Fisheries Service, National Oceanographic and Atmospheric Administration8901 La Jolla Shores Dr., La Jolla, California, 92037; 3 British Antarctic Survey, Natural Environment Research CouncilHigh Cross, Madingley Road, Cambridge, CB 0ET, U.K; 4 Marine Biology Research Division, Scripps Institution of Oceanography, University of California San Diego9500 Gilman Dr., La Jolla, California, 92093-0202; 5 Department of Animal Behaviour, University of BielefeldPostfach 100131, 33501, Bielefeld, Germany

**Keywords:** Colonization, gene flow, genetic differentiation, genetic diversity, pinniped

## Abstract

Many species have been heavily exploited by man leading to local extirpations, yet few studies have attempted to unravel subsequent recolonization histories. This has led to a significant gap in our knowledge of the long-term effects of exploitation on the amount and structure of contemporary genetic variation, with important implications for conservation. The Antarctic fur seal provides an interesting case in point, having been virtually exterminated in the nineteenth century but subsequently staged a dramatic recovery to recolonize much of its original range. Consequently, we evaluated the hypothesis that South Georgia (SG), where a few million seals currently breed, was the main source of immigrants to other locations including Livingston Island (LI), by genotyping 366 individuals from these two populations at 17 microsatellite loci and sequencing a 263 bp fragment of the mitochondrial hypervariable region 1. Contrary to expectations, we found highly significant genetic differences at both types of marker, with 51% of LI individuals carrying haplotypes that were not observed in 246 animals from SG. Moreover, the youngest of three sequentially founded colonies at LI showed greater similarity to SG at mitochondrial DNA than microsatellites, implying temporal and sex-specific variation in recolonization. Our findings emphasize the importance of relict populations and provide insights into the mechanisms by which severely depleted populations can recover while maintaining surprisingly high levels of genetic diversity.

## Introduction

Recently established populations may experience rapid genetic divergence, a process often attributed to founder effects (Leblois and Slatkin 2007). This occurs because isolated populations established by small numbers of founders tend not only to carry low genetic diversity but also to experience accelerated genetic drift. Classical examples of founder effects accompanying genetic bottlenecks are provided by studies of small and isolated mammalian populations such as bighorn sheep (Hedrick et al. 2001) and gray wolves (Liberg et al. 2005). However, processes that can lead to rapid genetic differentiation in larger, continuous populations remain poorly understood. This is especially true of species that are highly vagile and long-lived, as high mobility will tend to undermine population structure while long generation times slow the effective rate of genetic drift.

Rapid genetic changes have previously been observed in bird species introduced into geographic regions that lie beyond their normal ranges (e.g. Baker and Moeed 1987; Baker et al. 1990). However, in such cases it can be difficult to dissect apart the relative contributions of drift and selection, as many introductions involve “alien” habitats that may differ both ecologically and climatically from those experienced normally (Baker and Moeed 1987). Moreover, relatively little is known about alternative scenarios such as anthropogenic exploitation, which also hold the potential to bring about rapid and profound genetic alteration.

Populations of many pinniped species, in particular fur seals and sea lions, have been dramatically reduced by hunters, yet have managed to rebound (Gerber and Hilborn 2001) providing ideal case studies for exploring the impact of historical exploitation on contemporary patterns of genetic diversity and population structure. Pinnipeds are also interesting because, on the one hand, most species are long-lived (adults can live for 15–25 years; Riedman 1990) and are able to disperse and breed across long distances (Fabiani et al. 2003), factors that tend to undermine the formation of population structure. On the other hand, some species show female natal philopatry and both genders can be highly faithful to breeding sites (e.g. Pomeroy et al. 2000), promoting genetic differentiation (Matthiopoulos et al. 2005). Thus, population genetic structure will depend critically on the interplay of these behavioral and life-history traits.

The Antarctic fur seal, *Arctocephalus* (*Arctophoca*) *gazella*, is a typical pinniped species, being highly polygynous (Hoffman et al. 2003) and breeding in densely crowded colonies to which females show natal philopatry and both sexes show strong breeding site fidelity (Hoffman et al. 2006; Hoffman and Forcada 2011). Although it is difficult to objectively quantify the longevity of adult males, females can live for more than 20 years (Forcada and Staniland 2009) and have an average generation time of roughly a decade (9.89 ± 2.42 years, range = 4.83–12.72; Forcada et al. 2008). Moreover, this species is also highly vagile, as indicated by sightings of individuals as far afield as Brazil, South Africa, and even Australia (IUCN Red List, http://www.iucnredlist.org).

Like many other members of the *Arctocephalus* genus, Antarctic fur seals were subject to uncontrolled exploitation for their fur and oil during the early nineteenth century. At South Georgia (SG), sealing began in 1786 and it was estimated that by 1822, up to 1.2 million seals had been taken (Weddell 1825). Sealing in SG collapsed by 1885–1886, when two expeditions reported that only one and three seals had been sighted on the island. Subsequently, scattered sealing efforts seem to have eliminated any incipient population growth (Bonner 1968). Hunting ceased by 1907, and by then, the population was considered virtually extirpated. Using genetic data, Hoffman et al. (2011) estimated an *N*_*e bottleneck*_ of <500 individuals approximately 100 years ago, which is highly congruent with historical accounts. The SG population showed no signs of recovery until the 1930s, but numbers rebounded over the following five to six decades. It is now estimated to be in excess of 3 million (Hofmeyr et al. 2005), corresponding to around 97% of the global population.

A similar process unfolded at Livingston Island (LI) in the South Shetland archipelago, with intense sealing activities from 1822 to 1825 leading to local extirpation (McCann and Doidge 1987). Fur seals were not observed again until 1958, when 27 and 15 seals were seen ashore at the north and northwest corner of Cape Shirreff, respectively (LI; O'Gorman 1961). Within four decades, the population recovered from approximately 50 individuals (1966 census by Aguayo and Torres 1967) to over 20,000 (Hucke-Gaete et al. 2004). During its peak (1965–1973), the population growth rate at LI, estimated at 58%, was considered unattainable by intrinsic processes alone, and was therefore attributed to immigration from the already large and expanding SG population (Hucke-Gaete et al. 2004).

Despite the Antarctic fur seal having experienced drastic population reductions, only a single genetic study has so far examined this species' global population structure (Wynen et al. 2000). Genetic differentiation was reported to be overall weak on the basis of mitochondrial DNA (mtDNA), although two distinct mitochondrial clades were recognized, one comprising SG, the South Shetlands, Bouvet, and Marion Island, and a second comprising the eastern populations of Iles Kerguelen and Macquarie Island. No significant genetic difference was found (pairwise Φ_ST_) between SG and the South Shetland Islands. Wynen et al. (2000) also documented several haplotypes that were unique to some of the smaller fur seal populations. Although their sample sizes were too small to draw firm conclusions (*n* ≤ 20 per population), the authors interpreted the absence of certain haplotypes from SG as meaning that at least some contemporary fur seal populations may have been founded from more than one source. This merits further exploration, as being able to reliably exclude SG as the main source of fur seal immigrants to these populations would have important implications for understanding modes of recolonization that allow long-lived and highly mobile species to maintain high levels of genetic diversity despite dramatic historical reductions in population size.

Here, we used a large sample of 366 fur seal individuals to document genetic relationships between LI on the South Shetland Islands and its main putative source population within the western region, SG. To provide both matrilineal and biparental perspectives, all individuals were sequenced at a 263 bp fragment of the mitochondrial hypervariable region 1 (HVR1) and genotyped at 17 highly polymorphic microsatellite loci. We also added a fine-scale perspective by sampling three populations at LI that were successively established during the late twentieth century. Our aims were to evaluate support for the hypothesis that fur seal colonies at LI were mainly established by individuals from SG, and to test for genetic differences among the three colonies at LI that could be indicative of subtle differences in their recolonization histories.

## Methods

### Study sites and sample collection

**Figure 1:**
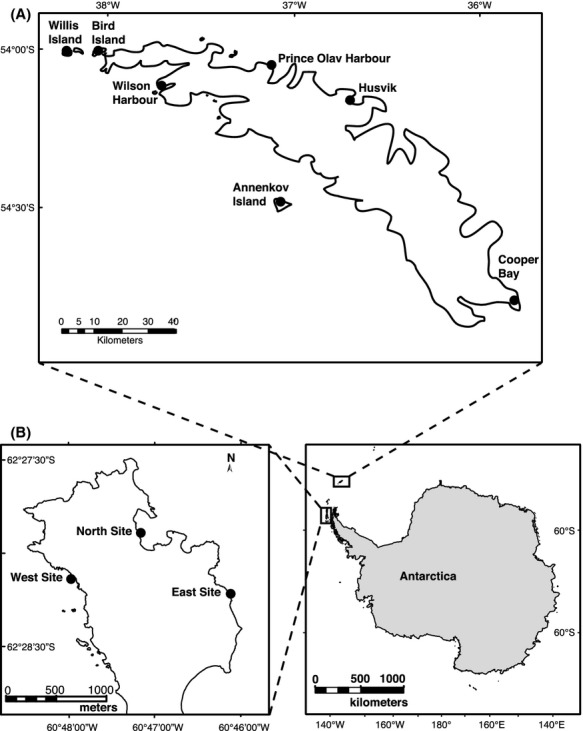
The sub-Antarctic and Antarctic islands of South Georgia and Livingston, where Antarctic fur seals were sampled. (A) South Georgia sampling sites; (B) Livingston Island sampling sites.

SG (35°47′–38°01′W and 53°58′–54°53′S) is a sub-Antarctic island situated approximately 1000 km southeast of the Falkland Islands (Fig. 1). Antarctic fur seal pups were tissue sampled by Hoffman et al. (2011) at seven sampling locations during the austral summer of 2003–2004 (Table 1). LI is the southernmost Antarctic fur seal breeding area and is one of the South Shetland Islands, a 500-km-long archipelago toward the north of the Antarctic Peninsula (Fig. 1). Sampling was conducted at Cape Shirreff (62°27′S; 60°47′W), an ice-free peninsula approximately 3 km long and located at the western end of LI's north coast.

**Table 1:** Number of Antarctic fur seals, *Arctocephalus gazella*, sampled at South Georgia and Livingston Island

Region	Sampling site	Samples sequenced at 263 bp of mtDNA	Samples genotyped at 17 microsatellites
Livingston Island	East	26	28
	West	46	43
	North	47	49
Subtotal		119	120
South Georgia	Willis Islands	16	15
	Bird Island	167	171
	Prince Olav	12	12
	Leith Harbor, Husvik	13	11
	Cooper Bay	14	14
	AnnenKov Island	15	14
	Wilson Harbor	9	9
Subtotal		246*	246
Total		365	366

* Sequences from South Georgia previously published by Hoffman et al. (2011).

Cape Shirreff fur seal pups were sampled at three sites (West, East, and North; hereafter designated LI-W, LI-E, and LI-N, respectively; Fig. 1, Table 1). LI-W is the oldest breeding site where the first records of fur seals were collected in the late 1950s (O'Gorman 1961). LI-N was recolonized in the 1980s, whereas LI-E is the most recently established breeding area, dating to 2001–2002. Samples were collected during the austral summers of 2008–2009 at LI-E, and 2009–10 at LI-W and LI-N.

Tissue samples were preserved in either 20% dimethylsulfoxide (DMSO) saturated with salt (NaCl), or 95% ethanol (ETOH) stored at −20°C. Total genomic DNA was subsequently extracted from LI tissue samples using a NaCl precipitation method (Miller et al. 1988). SG samples were extracted using either a Chelex 100 protocol (Walsh et al. 1991) for DNA used in sequencing, or a Dneasy blood and tissue extraction kit (Qiagen, http://www.qiagen.com/About-Us/Who-We-Are/) for DNA used in genotyping.

### Mitochondrial DNA sequencing

A 316 bp HVR1 fragment was polymerase chain reaction **(**PCR) amplified using the primers Thr/Pro (5′-TCCCTAAGACTCAAGGAAGAG-3′) and Cent (5′-GAGC GAGAAGAGGTACACTTT-3′) as detailed by Wynen et al. (2000) and Hoffman et al. (2011). Sequencing was initially carried out using the forward primer, but whenever sequences had <100% quality scores (as was the case for 24 of the 119 LI samples) the reverse strand was also sequenced. In addition, 24 randomly selected samples were independently replicated for quality control purposes, but no errors were detected. Sequences were edited using SEQUENCHER v. 4.8 for Windows (GeneCodes Corporation©, Ann Arbor, MI). The sequences were then trimmed to the final length of 263 bp following Hoffman et al. (2011) to eliminate insertions and deletions, including the highly repetitive “TC landmark” previously described by Wynen et al. (2000). Alignment was conducted using BIOEDIT v. 5.0.6 (Hall 1999).

### Microsatellite genotyping

Tissue samples previously genotyped by Hoffman et al. (2011) were transported to La Jolla, CA, where they were re-extracted and genotyped in the same laboratory where the LI samples were processed (Southwest Fisheries Science Center, National Oceanographic and Atmospheric Administration). This was done in order to assure that the genotype data for the two regions would be directly comparable.

All samples were genotyped at 17 microsatellite markers: Ag10 (Hoffman et al. 2008), Agaz8, Agaz9 (Hoffman 2009); Hl4, Hl16, Lc28 (Davis et al. 2002); Hg3.7 (Gemmell et al. 1997); M11A, M2B (Hoelzel et al. 1999); Pvc29, Pvc78 (Coltman et al. 1996); ZcCgDh1.8, ZcCgDh4.7, ZcCgDh48, ZcCgDh5.8, ZcCgDh7tg and ZcCgDhB.14 (Hernandez-Velazquez et al. 2005) using the annealing temperatures shown in Table S1. PCR amplification and fragment analysis protocols are described in detail elsewhere (Bonin et al. 2012). Following Hoffman and Amos (2005), we also independently regenotyped eight samples (2.2% of the samples) at all 17 loci. The resulting genotyping error rate was low at 0.02 per reaction (averaged across all loci), consistent with a previously published rate for a similar marker panel in the same laboratory (Bonin et al. 2012).

### Mitochondrial sequence analysis

Molecular diversity indices for the data set including haplotype (gene) diversity, the number of polymorphic sites (S), nucleotide diversity (π), and the average number of nucleotide differences (k) were assessed using DNAsp v. 5.10.01 (Librado and Rozas 2009). Genetic differentiation was estimated using Φ statistics within a hierarchical analysis of molecular variance (AMOVA; Excoffier et al. 1992) framework in the program ARLEQUIN v. 3.5.1.2 (Excoffier and Lischer 2010). The hierarchical levels corresponded to tests at the individual level (within sites), among the 10 sampling sites and between the two regions: LI (three sites) and SG (10 sites). Statistical significance was determined using 1000 permutations of the data set. A median-joining network (MJ) of the mtDNA haplotypes was constructed using NETWORK v. 4.6.1 (Bandelt et al. 1999).

Lastly, the total number of haplotypes at SG and LI was estimated to assess potential biases caused by incomplete haplotype sampling. We employed Dixon's method (Dixon 2006), which uses Bayes' Theorem to calculate a probability for the total number of haplotypes (*n* sampled and unsampled) given the number of observations and number of haplotypes sampled in a population. In order to obtain accurate estimates of variance, the analysis was set to increase *n* until its probability dropped below a 1/10^10^ proportion of the highest probability.

### Microsatellite data analysis

The microsatellite data set was tested for deviations from Hardy–Weinberg equilibrium (heterozygote deficit) and linkage disequilibrium using 100,000 dememorizations and 10,000 iterations per batch within GENEPOP v. 4.0 (Raymond and Rousset 1995). Null allele frequencies were estimated using CERVUS v. 3.0.3 (Marshall et al. 1998). Note that our data set comprises pups only, which were sampled at random within seasons at each of the sites. This sampling protocol minimized as far as is practicably possible the chance of sampling closely related individuals within seasons because female fur seals almost always give birth to a single pup per season. Nevertheless, to mitigate any potential concerns over the presence of closely related individuals such as full siblings within the data set, which could bias the assessment of genetic structure (Rodríguez-Ramilo and Wang 2012), we estimated pairwise relatedness values (*r*_xy_) for all individuals within SG and LI using COANCESTRY v. 1.0 (Wang 2011) according to Milligan's algorithm (Milligan 2003).

FSTAT v.2.9.3.2 (Goudet 1995) was next used to estimate variance components within individuals, among individuals within sampling sites and among sampling sites. Genetic differentiation was quantified by calculating global and pairwise *F*_ST_ values (θ; Weir and Cockerham 1984). Allelic richness (overall samples) and expected and observed heterozygosities (*H*_*e*_ and *H*_*o*_) were calculated according to Nei (1987) within FSTAT and were compared among populations using two-tailed, sample size-weighted statistical tests based on 10,000 permutations of the data set.

For comparison, we also analyzed our data within STRUCTURE v. 2.3.4 (Pritchard et al. 2000). Detection of the true number of clusters (*K*) based solely on the log probability of data (Ln[Pr(x|*K*)]) is not always straightforward within STRUCTURE, particularly where population structure is weak or follows an isolation-by-distance pattern. Consequently, we applied the ad hoc statistical method of Evanno et al. (2005), which focuses on the rate of change in the log probability of data between successive *K* values. A conspicuous “jump” or increase in the log probability of data (equivalent to the highest Δ*K*) indicates the uppermost hierarchical number of clusters present in the data set. We initially ran STRUCTURE without a priori sampling location information, but later repeated the same analyses incorporating location information by setting LOCPRIOR to 1. All analyses were conducted using the following parameters: admixture, allele frequencies correlated, 10,000 burn-in period and 100,000 Markov chain Monte Carlo (MCMC) repetitions (following recommendations in the STRUCTURE user's manual). We conducted five independent runs for *K* = 1–10 and used STRUCTURE HARVESTER web core (Earl and vonHoldt 2012) to interpret the resulting outputs.

### Detection of recent migrants

Maximum likelihood methods as implemented in the program MIGRATE can be powerful for exploring migration rates among populations or subpopulations. However, these approaches can be strongly affected by unsampled or “ghost” populations (Slatkin 2005). Having only been able to sample animals from two of several globally distributed Antarctic fur seal populations, we therefore chose the alternative approach of Rannala and Mountain (1997) to detect individuals with recent migrant ancestry (i.e. to a maximum of two generations back). This derives the probability distribution of allele frequencies in each population using a Bayesian approach and then calculates assignment probabilities for each individual via comparison against those distributions. This tends to work well even when populations are only weakly differentiated, although power decreases as migrant ancestry goes back in time across generations. We implemented this analysis within GENECLASS2 (Piry et al. 2004) using Rannala and Mountain's Bayesian criterion and the simulation algorithm proposed by Paetkau et al. (2004). MCMC resampling was performed with 10,000 simulated individuals and a *P*-value threshold of 0.01. In order to verify the robustness of GENECLASS2 results, we also used STRUCTURE to identify individuals with recent migrant ancestry. We set up migrant detection runs in STRUCTURE with the same parameters and run lengths described earlier. Three independent runs were performed to detect migrant descendants only within two generations (GENSBACK= 2) for each of three alternative migration model priors (MIGPRIOR= 0.01, 0.03, and 0.05).

## Results

### Mitochondrial DNA sequences

A total of 52 polymorphic sites and 41 haplotypes were observed among the 365 HVR1 mtDNA sequences. Thirteen haplotypes were only observed in SG (*n* = 246 individuals), five of which were represented by more than one individual. Fifteen haplotypes were unique to LI (*n* = 119 individuals), 10 of which were sampled more than once. Remarkably, unique regional haplotypes were found in 51% of the individuals sampled at LI, with the highest incidence being observed at the oldest colony (54%, LI-W), the lowest at the youngest colony (38%, LI-E), and an intermediate proportion at the colony of intermediary age (46%, LI-N).

Approximately 95% of the variation in the sequence data was observed among individuals within sampling locations (AMOVA, Φ_ST_ = 0.048, *P* = 0.00098 ± 0.0098), whereas the remaining 5% was largely partitioned between SG and LI (Φ_CT_ = 0.050, *P* = 0.00880 ± 0.00288). A negligible proportion of the total variance was explained by sampling sites within these two regions (Φ_SC_ = −0.001, *P* = 0.53,177 ± 0.01354). Consistent with this pattern, most of the significant pairwise Φ_ST_ values (9 of 11 significant values, *P* < 0.05; Table S2) were observed in comparisons between SG and LI. Sequence diversity indices were comparable between SG and LI (Table 2) despite the former having a far larger population size.

**Table 2:** Molecular diversity indices for Antarctic fur seals, *Arctocephalus gazella*, sampled in two regions (South Georgia and Livingston Island) sequenced for a 263 bp fragment (HVR1) of mtDNA and genotyped using 17 microsatellite markers

Marker type	Molecular diversity indices	South Georgia	Livingston Island
mtDNA	Number of individuals sequenced	246	119
	Number of unique haplotypes	13	15
	Average number of nucleotide differences	9.02	9.019
	Nucleotide diversity	0.034	0.034
Microsatellites	Number of individuals genotyped	246	120
	Mean number of alleles	11.824 ± 4.94	12.588 ± 5.26
	Allelic richness	6.021	6.343
	Mean heterozygote proportion	0.799 ± 0.115	0.802 ± 0.086
	Mean Nei's genic diversity	0.807 ± 0.104	0.822 ± 0.08

**Figure 2:**
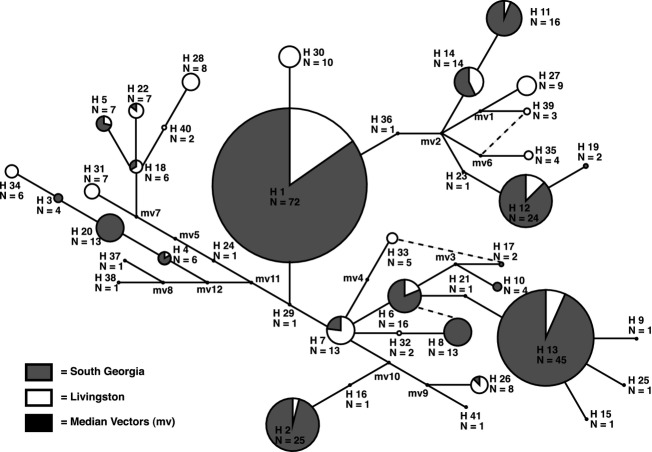
Median-joining network of 41 haplotypes observed among 365 Antarctic fur seals sampled at South Georgia and Livingston Island and sequenced for 263 bp fragment of the mtDNA control region (HVR1). Dashed lines represent unresolved links among haplotypes.

A MJ network constructed using all the samples contained 12 hypothetical median vectors (unsampled sequences) and three unresolved links (loops) despite attempts to reduce its complexity using postprocessing calculations within the program NETWORK. Nevertheless, Figure 2 shows that many of the most common haplotypes were present in both SG and LI, whereas haplotypes unique to LI tended to occupy peripheral positions in the network.

Analyses conducted to estimate the total number of haplotypes revealed that our sampling thoroughly encompassed haplotype diversity at both study areas, particularly SG. A total of 26 haplotypes (*P*(*n*) = 0.99) were estimated for SG (μ = 26.002; σ^2^ = 0.002; 95% CI: 26–26), which corresponds to the exact number of observed haplotypes. At LI, the number of estimated and sampled haplotypes was equivalent (*n* = 28), but this number had a lower probability (*P*(*n*) = 0.62) and higher variance (μ = 28.488; σ^2^ = 0.537; 95% CI: 28–30) suggesting a greater probability of missed haplotypes.

### Microsatellites

Our microsatellite panel was highly informative (average number of alleles per locus = 13.76 ± 6.95; *H*_*E*_ = 0.81) and the proportion of missing data was low at 1.6%. There was no clear indication of null alleles, allelic dropout, or linkage disequilibrium (Table S1). Four loci deviated significantly from Hardy–Weinberg equilibrium, although only two of these values remained significant following Bonferroni correction for multiple statistical tests. Moreover, these loci were not found to be consistently out of equilibrium when the data were analyzed separately for SG and LI, suggesting that these deviations could be due to a Wahlund effect (i.e. heterozygosity reduction due to population substructure). Analysis using COANCESTRY revealed a relatedness coefficient (*r*_xy_) distribution centered tightly around a mean of zero at both SG and LI. Only two of 30,135 pairwise comparisons at SG and four of 7140 pairwise comparisons at LI yielded *r*_xy_ values ≥0.50, suggesting a negligible effect of sampling kin.

The global *F*_ST_ (θ) for the microsatellite data set was 0.014 (95% CI = 0.010–0.018; 99% CI = 0.009–0.019). Pairwise *F*_ST_ values among sampling sites were mostly significant in comparisons involving SG and LI (23 of 24 inter-region comparisons; Table S3). A majority of nonsignificant, low pairwise *F*_ST_ values were indicative of a lack of genetic structure within SG (overall *F*_ST_ = 0.001 ± 0.006; range = −0.009–0.018). At LI, a similar overall result was obtained (overall *F*_ST_ = 0.008 ± 0.003; range = 0.005–0.010) with only comparisons involving the youngest colony (LI-E) reaching statistical significance (LI-E vs. LI-W, *F*_ST_ = 0.009; LI-E vs. LI-N, *F*_ST_ = 0.013, *P* < 0.001). Allelic richness and mean observed (*H*o) and expected (*H*s) heterozygosities did not differ significantly between SG and LI (*P* = 0.196, 0.803, and 0.170, respectively, in two-tailed comparisons).

**Figure 3:**
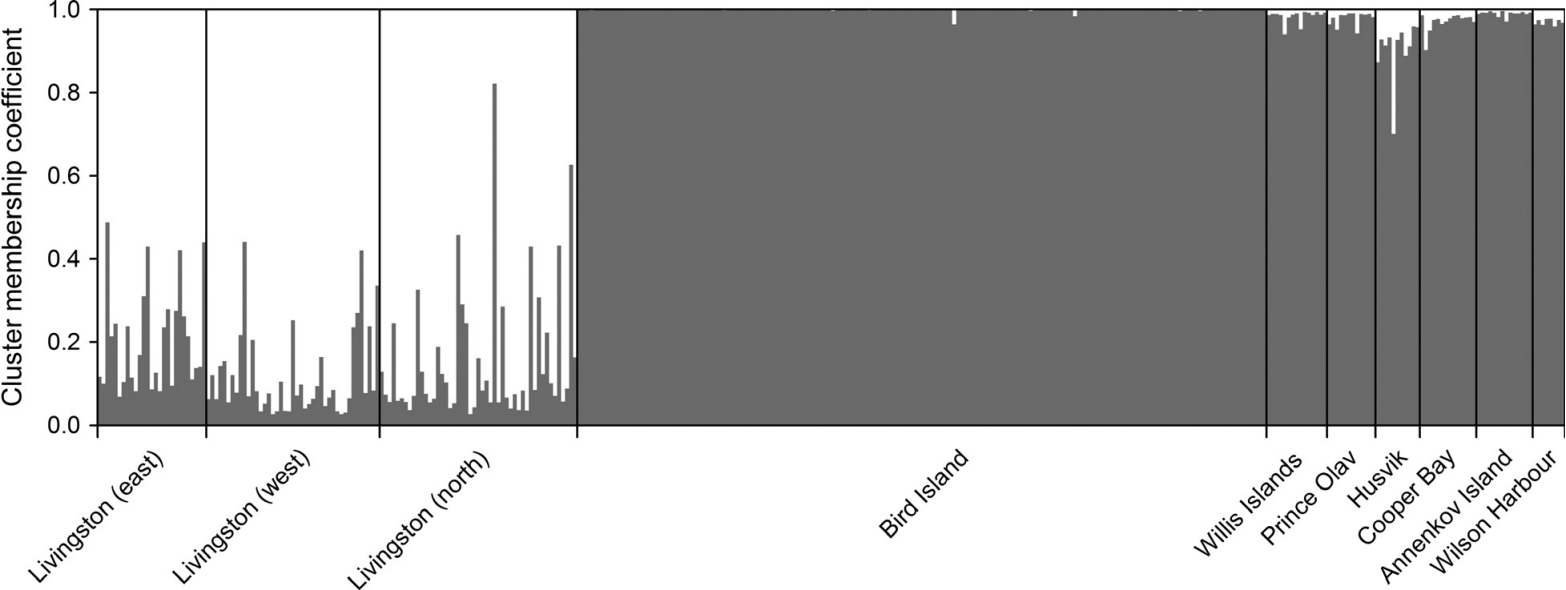
Posterior probability of assignment for Antarctic fur seal individuals (vertical bars) into clusters according to Bayesian analyses in STRUCTURE v.2.3.3 (Pritchard et al. 2000). Clusters corresponding to South Georgia and Livingston Island regions are denoted by dark and light gray, respectively (results shown incorporate sampling locations of individuals).

Consistent with the above analyses, STRUCTURE identified two clusters (*K* = 2) based on the approach of Evanno et al. (2005) (Fig. S1). These coincided perfectly with SG and LI, with the majority of individuals having a high posterior probability of assignment to their respective cluster (minimum of 90% for SG and 79% for LI individuals). Similar results were obtained using the LOCPRIOR setting, which takes into account the sampling locations of each individual (Fig. 3). Additional STRUCTURE runs conducted separately for LI and SG found no clear evidence of further subdivision within these two regions (results not shown).

### Detection of individuals with migrant ancestry

The program GENECLASS2 detected three pups with migrant ancestry via exclusion tests within LI-N (*P* = 0.0007, 0.0077, and 0.0041, respectively). Two of these were assigned to SG with >99.5% probability, whereas the third individual was not confidently assigned to either SG or LI, suggesting that it could have originated from another, unsampled location. The program STRUCTURE identified one of the same migrants using a migration prior of 0.01 and confirmed the second migrant with a higher migration prior of 0.05, while assignment probabilities to the population of origin (LI) were 0.004 (*P* < 0.0001) and 0.391 (*P* < 0.01), respectively.

## Discussion

We explored the recolonization history of an important top predator in the Southern Ocean, the Antarctic fur seal, by conducting a genetic analysis of pups sampled from LI and its main putative source population SG. We found highly significant differences in microsatellite allele frequencies and identified numerous mitochondrial haplotypes that were unique to LI, allowing us to reject a simple scenario of recolonization from SG. Our findings have important implications for understanding how severely depleted populations of long-lived mammals can maintain high levels of genetic diversity.

Our results are difficult to reconcile with the original working hypothesis that LI was mainly recolonized by immigrants from the rapidly expanding population of SG. First, significant genetic differences between LI and SG were observed in both nuclear and mitochondrial genomes, suggesting that the overall pattern of genetic differentiation is robust and not simply driven by, for example, female natal philopatry. Second, to be consistent with our data, a scenario of recolonization from SG would need to invoke a strong founder effect and at the same time require the colonists from SG to have carried mtDNA haplotypes that are so infrequent as not to be observable within our large sample of 246 individuals from SG. The latter seems improbable given that we sampled pups from most of the main breeding colonies around SG. This assumption was strongly corroborated by the fact that the estimated total number of haplotypes at SG was not greater than the observed number of haplotypes in our sample, indicating that our sampling allowed for a thorough inventory of haplotype diversity. Moreover, founder effects tend to be associated with reduced allelic richness (Allendorf and Luikart 2007), but we found that SG and LI had comparably high levels of genetic diversity. This is surprising given that historical population sizes at SG have been consistently much larger than LI. Although preharvesting data on these populations are limited, a rough estimate suggests that the preharvesting breeding population of *A. gazella* at LI might have been ca. 167,000 animals (Hucke-Gaete et al. 2004). A much larger population bred at SG, since historical accounts report that at least 1.2 million seals had been taken there by 1822 (Weddell 1825). Third, LI was recolonized only a few decades ago and female fur seals have a generation time of roughly a decade. Consequently, there has been very little time for intrinsic evolutionary processes such as genetic drift to operate.

Arguably a more likely explanation of our findings could be that Antarctic fur seals survived sealing in sufficient numbers at isolated locations within the South Shetland Islands archipelago to allow the nearby vacant rookeries at LI to be recolonized. This is plausible because, although the steepest phase of growth of the South Shetlands population has been largely attributed to immigration (Hucke-Gaete et al. 2004), systematic censuses incorporating all breeding areas did not commence until 1987 (Bengtson et al. 1990; Hucke-Gaete et al. 2004). Thus, relict populations in less accessible locations may well have been overlooked. The strongest contender would be the San Telmo Islets, which are adjacent to LI. A census held at San Telmo in 1987 estimated a total of 5781 seals, which at time was twice the size of the nearby Cape Shirreff population (Bengtson et al. 1990). However, by 1992 the Cape Shirreff seal population had surpassed San Telmo's and has remained larger ever since (Hucke-Gaete et al. 2004).

It is also possible that LI was recolonized by immigrants from one or more source populations from further afield. The best candidate for such a population within the “western region” proposed by Wynen et al. (2000) is Bouvet Island. This species may not have been completely exterminated at Bouvet, which currently holds the World's second largest Antarctic fur seal population (Hofmeyr et al. 2005). Other islands within the western region are less likely to have been significant sources of immigrants as their pup production is much lower, in most cases less than 400 and not more than 1000 pups per year (Hofmeyr et al. 1997, 2005; Page et al. 2003; Waluda et al. 2010). However, to determine the relative contributions, if any, of populations such as Bouvet Island would require allele frequency data from multiple colonies, most of which are remote and rarely visited.

As initially reported for SG (Hoffman et al. 2011), we also found little evidence for genetic structuring within LI, although contrasting results were obtained for mtDNA and microsatellites with respect to the newest colony, LI-E. Individuals from this locality were found to cluster together with those from LI based on the microsatellite data, but showed greater similarity to SG than the other two LI colonies based on mtDNA. By implication, many of the females who founded LI-E may have originated from SG, whereas the males they mated with could have been of local origin. This interpretation should be treated with caution because, although we sampled all the pups born at LI-E, the sample size for this colony is small (*n* = 26). Repeating the analysis after randomly selecting the same number of individuals from LI-W and LI-N, the genetic differences within LI became no longer significant. However, the latter analysis is highly conservative and it would be worthwhile collecting more samples from these three colonies in the future to explore this phenomenon in greater detail.

We also found evidence for at least two pups from LI having immigrant ancestry from SG within the last two generations. Although we were not able to formally estimate migration rates within a maximum likelihood framework due to incomplete population sampling, this provides evidence in support of some level of contemporary gene flow between SG and LI, primarily directed toward the more recently founded LI-E colony. This makes sense because the SG population reached carrying capacity fairly recently and may thus be spilling over into relatively nearby, lower density sites. In fact, Boyd (1993) suggested that emigration was the reason for a detectable decline in the annual increase in the SG population in the early 1990s, a consequence of overcrowding at traditional breeding beaches in SG (e.g. Bird Island).

Our results are interesting in a broader context, partly because very few genetic studies have explored the impacts of historical exploitation on long-lived vertebrate species, but also because those that have done so have reported little or no population structure. For example, Australian and Northern fur seals were both found to be panmictic despite these species having also been historically harvested (Dickerson et al. 2010; Lancaster et al. 2010). In both cases, genetic resolution may have been limited due to the use of five and seven microsatellite loci, respectively, in comparison to our 17. However, it also seems likely that higher contemporary migration rates and, in the case of the Australian fur seal, closer geographic proximity of colonies could have played a role.

In conclusion, our findings strongly support the hypothesis that LI was not simply recolonized from SG and instead point toward a more complex recolonization history in which the genetic contribution of SG may have varied both temporally and by sex. Our results also highlight the importance of relict populations, which although demographically less significant, can harbor unexpectedly high levels of genetic diversity. Such populations could become increasingly important for maintaining the diversity of polar species that are facing mounting threats from rapid environmental change.

## Data Accessibility

All tissue samples used in this study are currently housed at the Marine Mammal and Turtle Molecular Research Collection, Southwest Fisheries Science Center, National Oceanographic and Atmospheric Administration, La Jolla, CA, and the British Antarctic Survey, Cambridge, U.K. All raw genetic data can be found in a database managed by the Protected Resources Division at Southwest Fisheries Science Center, National Oceanographic and Atmospheric Administration, La Jolla, CA.

## Supporting Information

**Figure S1**. Results of Bayesian cluster analyses within the program STRUCTURE v.2.3.3 (Pritchard et al. 2000) based on 366 Antarctic fur seals genotyped for 17 microsatellite loci. Shown are plots of mean and standard deviation of the posterior probabilities of *K* (LnP(D)) plus variation in the rate of increase of LnP(D) with successive *K* values (ΔK). Five simulations were conducted for each value of *K* between one and ten. (A, B) Results of runs without a priori population information. (C, D) Results of runs with population information (sampling locations).

**Table S1.** Microsatellite loci used to genotype 366 Antarctic fur seal samples (*n* = 246 from South Georgia, *n* = 120 from Livingston Island).

**Table S2.** Pairwise Φ_ST_*s* (above diagonal) and corresponding *P-*values (below diagonal) estimated for 365 Antarctic fur seals, *Arctocephalus gazella*, sampled at 10 sites across two regions (South Georgia and Livingston Island) and sequenced for 316 bp of the mtDNA HVR1.

**Table S3.** Pairwise *F*_ST_s (θ, above diagonal) and corresponding *P-*values (below diagonal) estimated for 366 Antarctic fur seals, *Arctocephalus gazella*, sampled at 10 sites across two regions (South Georgia and Livingston Island) and genotyped at 17 microsatellite loci.
