# Supplementary material for: Unexpected genetic differentiation between recently recolonized populations of a long-lived and highly vagile marine mammal
Source: Ecol Evol. 2013 Sep 8;3(11):3701–12. doi: 10.1002/ece3.732 (PMC3810869; doi:10.1002/ece3.732)

**Supplementary Information**

**Table S1.** Microsatellite loci used to genotype 366 Antarctic fur seal samples (*n*= 246 from South Georgia, *n*= 120 from Livingston Island). The table includes details of PCR annealing temperatures (Tm), number of alleles (k), observed and expected heterozygosities (Ho and H_E_), null allele frequencies (F null) and Hardy-Weinberg equilibrium test p values for the total dataset (*p* global), for Livingston Island (*p* LI) and South Georgia samples (*p* SG). Significant deviations for Hardy-Weinberg equilibrium are highlighted in bold.

| **Locus** | **Source** | **Species** | **Tm** | **K** | **Ho** | **He** | **F (null)** | **p Global** | **p LI** | **p SG** |
| --- | --- | --- | --- | --- | --- | --- | --- | --- | --- | --- |
| Ag10 t | Hoffman et al., 2007 | *Arctocephalus gazella* | 50 | 8 | 0.751 | 0.761 | 0.0139 | 0.568 | 0.754 | 0.508 |
| Agaz8t | Hoffman et al., 2009 | *Arctocephalus gazella* | 52 | 19 | 0.838 | 0.864 | 0.0134 | **0.000** | 0.103 | **0.001** |
| Agaz9t | Hoffman et al., 2009 | *Arctocephalus gazella* | 50 | 10 | 0.775 | 0.807 | 0.0231 | 0.098 | 0.072 | 0.244 |
| Hg3.7 t | Gemmell et al., 1997 | *Halichoerus grypus* | 50 | 13 | 0.833 | 0.853 | 0.0071 | 0.004 | 0.007 | 0.479 |
| Hl-16 t | Davis et al., 2002 | *Hydrurga leptonyx* | 56 | 35 | 0.864 | 0.887 | 0.0039 | 0.322 | 0.021 | 0.837 |
| HI-4 t | Davis et al., 2002 | *Hydrurga leptonyx* | 52 | 5 | 0.529 | 0.601 | 0.0648 | 0.008 | 0.051 | 0.003 |
| Lc-28 t | Davis et al., 2002 | *Lobodon carcinophaga* | 58 | 14 | 0.845 | 0.855 | 0.023 | **0.000** | 0.003 | 0.060 |
| M2B t | Hoelzel, 1999 | *Mirounga angustirostris* | 56 | 13 | 0.832 | 0.845 | 0.0245 | 0.027 | 0.740 | 0.014 |
| M11C t | Russ Hoelzel unpubl. | *Mirounga angustirostris* | 55 | 20 | 0.882 | 0.899 | 0.0065 | 0.148 | 0.006 | 0.892 |
| Pvc29 | Coltman et al., 1996 | *Phoca vitulina* | 52 | 15 | 0.869 | 0.865 | 0.0017 | 0.358 | 0.613 | 0.426 |
| Pvc78 | Coltman et al.,1996 | *Phoca vitulina* | 55 | 10 | 0.804 | 0.814 | 0.0078 | 0.283 | 0.551 | 0.269 |
| ZcCgDh1.8 t | Hernandez-Velazquez et al. 2005 | *Zalophus californianus* | 60 | 9 | 0.757 | 0.77 | 0.0092 | 0.125 | 0.327 | 0.390 |
| ZcCgDh4.7 t | Hernandez-Velazquez et al. 2005 | *Zalophus californianus* | 60 | 14 | 0.849 | 0.88 | 0.0119 | 0.257 | 0.073 | 0.688 |
| ZcCgDh48 t | Hernandez-Velazquez et al. 2005 | *Zalophus californianus* | 55 | 10 | 0.624 | 0.602 | -0.0288 | 0.870 | 0.657 | 0.885 |
| ZcCgDh5.8 | Hernandez-Velazquez et al. 2005 | *Zalophus californianus* | 60 | 15 | 0.886 | 0.881 | -0.001 | 0.288 | 0.671 | 0.546 |
| ZcCgDh7tg t | Hernandez-Velazquez et al. 2005 | *Zalophus californianus* | 55 | 18 | 0.907 | 0.889 | -0.0104 | 0.694 | 0.656 | 0.747 |
| ZcCgDhB.14 t | Hernandez-Velazquez et al. 2005 | *Zalophus californianus* | 60 | 6 | 0.762 | 0.771 | 0.012 | 0.022 | 0.285 | 0.046 |

**Table S2.** Pairwise Φ_ST_s (above diagonal) and corresponding *p* values (below diagonal) estimated for 365 Antarctic fur seals, *Arctocephalus gazella*, sampled at 10 sites across two regions (South Georgia and Livingston Island) and sequenced for 316 bp of the mtDNA HVR1. Statistically significant comparisons (*p* < 0.05) are highlighted in bold. Sample sizes are given in Table 1.

| **South Georgia** | | | | | | | | **Livingston Island** | | |
| --- | --- | --- | --- | --- | --- | --- | --- | --- | --- | --- |
|  | **Husvik** | **Wilson Harbor** | **Prince Olav** | **Cooper Bay** | **Annenkov** | **Willis Island** | **Bird Island** | **LI- East** | **LI- West** | **LI- North** |
| **Husvik** |  | -0.027 | 0.069 | 0.015 | 0.011 | 0.084 | 0.000 | -0.006 | 0.003 | 0.028 |
| **Wilson Harbor** | 0.636±0.004 |  | -0.009 | -0.044 | -0.061 | 0.045 | -0.019 | -0.027 | 0.018 | 0.016 |
| **Prince Olav** | **0.046±0.002** | 0.505±0.004 |  | 0.015 | 0.002 | -0.042 | 0.001 | 0.012 | 0.072 | 0.055 |
| **Cooper Bay** | 0.329±0.004 | 0.795±0.004 | 0.282±0.004 |  | -0.027 | 0.036 | -0.009 | 0.029 | 0.065 | 0.076 |
| **Annenkov** | 0.316±0.004 | 0.961±0.001 | 0.372±0.004 | 0.746±0.004 |  | 0.054 | 0.008 | -0.008 | 0.041 | 0.022 |
| **Willis Island** | **0.040±0.002** | 0.168±0.003 | 0.606±0.004 | 0.155±0.003 | 0.097±0.002 |  | 0.013 | 0.050 | 0.103 | 0.099 |
| **Bird Island** | 0.396±0.005 | 0.662±0.004 | 0.392±0.004 | 0.590±0.004 | 0.263±0.004 | 0.206±0.003 |  | 0.017 | 0.052 | 0.057 |
| **LI- East** | 0.526±0.004 | 0.799±0.003 | 0.263±0.004 | 0.117±0.003 | 0.599±0.004 | 0.054±0.002 | 0.102±0.002 |  | -0.007 | -0.016 |
| **LI- West** | 0.371±0.005 | 0.218±0.004 | **0.015±0.001** | **0.012±0.001** | **0.037±0.001** | **0.000±0.000** | **0.000±0.000** | 0.654±0.004 |  | -0.004 |
| **LI- North** | 0.108±0.003 | 0.236±0.004 | **0.033±0.001** | **0.004±0.000** | 0.124±0.002 | **0.001±0.000** | **0.000±0.000** | 0.932±0.002 | 0.587±0.004 |  |

**Table S3.** Pairwise F_ST_s (θ, above diagonal) and corresponding *p* values (below diagonal) estimated for 366 Antarctic fur seals, *Arctocephalus gazella*, sampled at 10 sites across two regions (South Georgia and Livingston Island) and genotyped at 17 microsatellite loci. Significant comparisons (*p* < 0.05) are highlighted in bold. Sample sizes are given in Table 1.

| **South Georgia** | | | | | | | | **Livingston Island** | | |
| --- | --- | --- | --- | --- | --- | --- | --- | --- | --- | --- |
|  | **Husvik** | **Wilson Harbor** | **Prince Olav** | **Cooper Bay** | **Annenkov** | **Willis Island** | **Bird Island** | **LI- East** | **LI- West** | **LI- North** |
| **Husvik** |  | 0.003 | 0.007 | -0.001 | 0.019 | 0.005 | 0.006 | 0.023 | 0.019 | 0.014 |
| **Wilson Harbor** | 0.353 |  | -0.002 | -0.001 | 0.007 | -0.003 | 0.001 | 0.016 | 0.014 | 0.017 |
| **Prince Olav** | 0.184 | 0.716 |  | -0.004 | 0.000 | -0.009 | 0.003 | 0.014 | 0.021 | 0.018 |
| **Cooper Bay** | 0.507 | 0.817 | 0.834 |  | 0.000 | -0.007 | -0.003 | 0.013 | 0.018 | 0.016 |
| **Annenkov** | **0.023** | 0.201 | 0.469 | 0.569 |  | -0.003 | 0.004 | 0.030 | 0.029 | 0.024 |
| **Willis Island** | 0.266 | 0.350 | 0.904 | 0.918 | 0.410 |  | -0.005 | 0.021 | 0.019 | 0.021 |
| **Bird Island** | 0.097 | 0.528 | 0.177 | 0.501 | 0.167 | 0.571 |  | 0.024 | 0.021 | 0.020 |
| **LI- East** | **<0.001** | **0.032** | **0.002** | **0.006** | **<0.001** | **<0.001** | **<0.001** |  | 0.009 | 0.013 |
| **LI- West** | **<0.001** | **0.003** | **<0.001** | **<0.001** | **<0.001** | **<0.001** | **<0.001** | **<0.001** |  | 0.005 |
| **LI- North** | **0.006** | **0.003** | **<0.001** | **<0.001** | **<0.001** | **<0.001** | **<0.001** | **<0.001** | 0.193 |  |

**Figure S1**. Results of Bayesian cluster analyses within the program STRUCTURE v.2.3.3 (Pritchard et al., 2000) based on 366 Antarctic fur seals genotyped for 17 microsatellite loci. Shown are plots of mean and standard deviation of the posterior probabilities of *K* (LnP(D)) plus variation in the rate of increase of LnP(D) with successive *K* values (ΔK). Five simulations were conducted for each value of *K* between one and ten. a, b) Results of runs without *a priori* population information. c, d) Results of runs with population information (sampling locations).


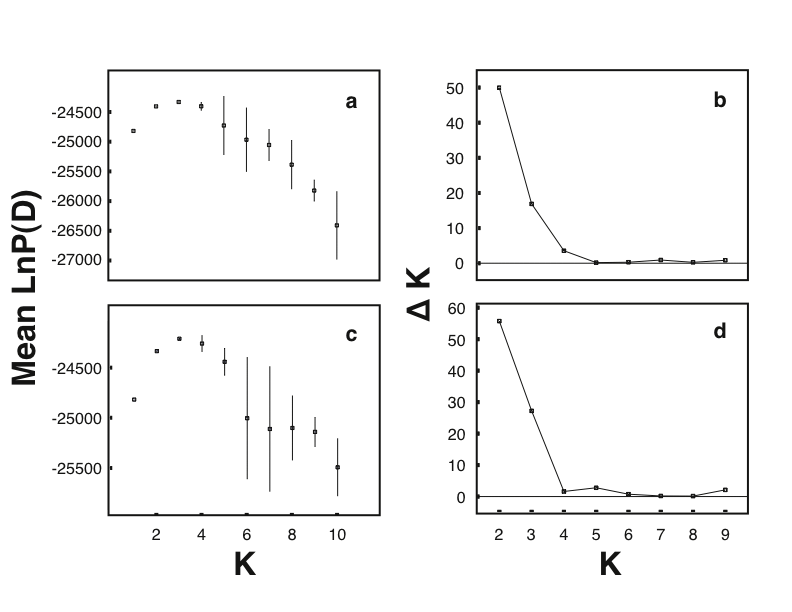

Supplement: Supplementary file 1 [file ece30003-3701-SD1.docx]
